# Supplementary material for: Does thinking make it so? Differential associations between adversity worries and experiences and mental health during the COVID-19 pandemic
Source: J Epidemiol Community Health. 2021 Jan 22:jech-2020-215598. doi: 10.1136/jech-2020-215598 (PMC7830321; doi:10.1136/jech-2020-215598)
Supplement: Supplementary file 1 [file jech-2020-215598supp001.pdf]

SUPPLEMENTARY INFORMATION

Participants
Participants were recruited using three primary approaches. First, snowballing was used, including promoting the study through existing networks and mailing lists (including large databases of adults who had previously consented to be involved in health research across the UK), print and digital media coverage, and social media. Second, more targeted recruitment was undertaken focusing on (i) individuals from a low-income background, (ii) individuals with no or few educational qualifications, and (iii) individuals who were unemployed. Third, the study was promoted via partnerships with third sector organisations to vulnerable groups, including adults with pre-existing mental health conditions, older adults, carers, and people experiencing domestic violence or abuse.

Analysis
The basic model for mental health (MH) can be expressed as follows:

$$MH_{it} = \beta_{0t} + \beta_1 E_{ikt} + \beta_2 W_{ikt} + \beta_3 D_t + \beta_4 N_t + \alpha_i + \varepsilon_{it}$$

where MH_{it} is a measure of individual i's anxiety and depression score at time t, E is whether an individual i was experiencing adversity k at time t, W is whether an individual i was worrying about adversity k at time t, Dt is a vector of indicator variables for day, Nt is a continuous variable for days since lockdown, α_i is unobserved time invariant confounding factors, and ε is error. Whether E_{ikt} or W_{ikt} were entered simultaneously or separately depends on the model.

In our sensitivity analyses, when using a continuous measure of SEP derived from a confirmatory factor analysis (CFA) of the five SEP indicator variables, the CFA used weighted least square mean and given the discrete nature of the SEP indicators, the variance adjusted (WLSMV) estimator was implemented. The RMSEA of the CFA model was 0.08, indicating an adequate fit [23]. We split the latent factor into quintiles using natural breaks in the factor values.

Tables & Figures

Table S1: Mean and standard deviations for study outcomes and exposures

	Variable	Overall Mean	Overall SD	Between SD	Within SD
Outcome	PHQ-9 Likert	6.26	5.99	5.65	1.99
	GAD-7 Likert	4.67	5.24	4.93	1.79
Experiences	Adversity experiences	0.60	0.84	0.80	0.28
	Lost work	0.10	0.30	0.29	0.08
	Cut in income	0.20	0.40	0.38	0.13
	Unable to access sufficient food	0.04	0.20	0.15	0.13
	Unable to access required medication	0.03	0.16	0.12	0.11
	Suspected or diagnosed COVID-19	0.14	0.34	0.33	0.08
	Physically or psychologically harmed	0.09	0.29	0.27	0.11
Worries	Adversity worries	1.30	1.32	1.14	0.65
	Losing job/unemployment	0.13	0.34	0.28	0.19
	Finances	0.31	0.46	0.39	0.24
	Getting food	0.19	0.40	0.30	0.26
	Getting medication	0.11	0.32	0.24	0.20
	Catching COVID-19	0.42	0.49	0.40	0.29
	Personal safety	0.13	0.34	0.25	0.22

Table S2: PHQ-9 and GAD-7 scores by SEP level, weighted figures.

Low SEP Index	n (%)	GAD-7	PHQ-9
0	15,521.8 (41.47%)	4.43 (4.77)	5.54 (5.19)
1	12,858.8 (34.35%)	5.11 (5.32)	6.72 (5.93)
2+	9,052.7 (24.18%)	6.78 (6.31)	9.17 (7.06)

Table S3: (Unweighted) sample characteristics by rounds of data collection in study period. "Excluded" includes participants with fewer than 2 rounds of data collection with complete data.

			Total Rounds of Data Collection				
	Variable	Excluded	2	3	4	5	6
	n	17,086 (28.96%)	7,516 (12.74%)	5,036 (8.54%)	6,577 (11.15%)	13,411 (22.73%)	9,369 (15.88%)
Age (grouped)	18-34	4,840 (28.33%)	1,559 (20.74%)	1,028 (20.41%)	939 (14.28%)	1,592 (11.87%)	1,218 (13%)
	35-49	6,085 (35.61%)	2,715 (36.12%)	1,674 (33.24%)	2,005 (30.49%)	3,908 (29.14%)	2,591 (27.66%)
	50-64	4,325 (25.31%)	2,249 (29.92%)	1,514 (30.06%)	2,176 (33.08%)	4,824 (35.97%)	3,297 (35.19%)
	65+	1,836 (10.75%)	993 (13.21%)	820 (16.28%)	1,457 (22.15%)	3,087 (23.02%)	2,263 (24.15%)
Country of residence	England	14,535 (85.07%)	6,289 (83.67%)	3,589 (71.27%)	4,427 (67.31%)	10,785 (80.42%)	8,234 (87.89%)
	Scotland	1,073 (6.28%)	408 (5.43%)	348 (6.91%)	414 (6.29%)	977 (7.29%)	688 (7.34%)
	Wales	1,243 (7.27%)	747 (9.94%)	1,032 (20.49%)	1,661 (25.25%)	1,509 (11.25%)	354 (3.78%)
	Northern Ireland	235 (1.38%)	72 (0.96%)	67 (1.33%)	75 (1.14%)	140 (1.04%)	93 (0.99%)
Ethnicity	White	15,687 (91.81%)	7,081 (94.21%)	4,785 (95.02%)	6,315 (96.02%)	12,908 (96.25%)	8,980 (95.85%)
	Non-White	1,399 (8.19%)	435 (5.79%)	251 (4.98%)	262 (3.98%)	503 (3.75%)	389 (4.15%)
Gender	Male	4,505 (26.37%)	1,713 (22.79%)	1,205 (23.93%)	1,811 (27.54%)	3,648 (27.2%)	2,334 (24.91%)
	Female	12,581 (73.63%)	5,803 (77.21%)	3,831 (76.07%)	4,766 (72.46%)	9,763 (72.8%)	7,035 (75.09%)
Household income	£90k+	2,006 (11.74%)	874 (11.63%)	528 (10.48%)	622 (9.46%)	1,384 (10.32%)	1,151 (12.29%)
	£60k - £90k	2,690 (15.74%)	1,241 (16.51%)	823 (16.34%)	991 (15.07%)	1,949 (14.53%)	1,553 (16.58%)
	£30k - £60k	5,729 (33.53%)	2,646 (35.2%)	1,766 (35.07%)	2,376 (36.13%)	4,600 (34.3%)	3,351 (35.77%)
	£16k - £30k	3,856 (22.57%)	1,685 (22.42%)	1,199 (23.81%)	1,642 (24.97%)	3,359 (25.05%)	2,149 (22.94%)
	<£16k	2,805 (16.42%)	1,070 (14.24%)	720 (14.3%)	946 (14.38%)	2,119 (15.8%)	1,165 (12.43%)
Marital status	Single	3,391 (19.85%)	1,288 (17.14%)	814 (16.16%)	930 (14.14%)	2,005 (14.95%)	1,542 (16.46%)
	Divorced/Widowed	1,857 (10.87%)	889 (11.83%)	621 (12.33%)	936 (14.23%)	1,952 (14.56%)	1,293 (13.8%)
	In relationship but living apart	1,394 (8.16%)	542 (7.21%)	377 (7.49%)	382 (5.81%)	739 (5.51%)	533 (5.69%)
	Cohabiting with partner	10,444 (61.13%)	4,797 (63.82%)	3,224 (64.02%)	4,329 (65.82%)	8,715 (64.98%)	6,001 (64.05%)
Overcrowded accommodation	Not Overcrowded	15,470 (90.54%)	7,037 (93.63%)	4,772 (94.76%)	6,325 (96.17%)	12,993 (96.88%)	9,106 (97.19%)
	Overcrowded	1,616 (9.46%)	479 (6.37%)	264 (5.24%)	252 (3.83%)	418 (3.12%)	263 (2.81%)
Highest qualification	Postgraduate	4,399 (25.75%)	1,975 (26.28%)	1,415 (28.1%)	1,816 (27.61%)	3,627 (27.04%)	3,060 (32.66%)
	Undergraduate	6,611 (38.69%)	3,138 (41.75%)	2,102 (41.74%)	2,723 (41.4%)	5,519 (41.15%)	3,832 (40.9%)

			Total Rounds of Data Collection				
	Variable	Excluded	2	3	4	5	6
	A-Level or Vocational	3,248 (19.01%)	1,358 (18.07%)	867 (17.22%)	1,160 (17.64%)	2,391 (17.83%)	1,420 (15.16%)
	GCSE or Lower	2,828 (16.55%)	1,045 (13.9%)	652 (12.95%)	878 (13.35%)	1,874 (13.97%)	1,057 (11.28%)
Employment status	Employed	12,646 (74.01%)	5,579 (74.23%)	3,555 (70.59%)	4,310 (65.53%)	8,625 (64.31%)	5,991 (63.94%)
	Inactive	4,143 (24.25%)	1,849 (24.6%)	1,425 (28.3%)	2,199 (33.43%)	4,635 (34.56%)	3,294 (35.16%)
	Unemployed	297 (1.74%)	88 (1.17%)	56 (1.11%)	68 (1.03%)	151 (1.13%)	84 (0.9%)
Household tenure	Own Outright	3,639 (21.3%)	2,052 (27.3%)	1,512 (30.02%)	2,426 (36.89%)	5,175 (38.59%)	3,820 (40.77%)
	Own Mortgage	6,955 (40.71%)	3,119 (41.5%)	2,032 (40.35%)	2,514 (38.22%)	4,932 (36.78%)	3,329 (35.53%)
	Rent	6,492 (38%)	2,345 (31.2%)	1,492 (29.63%)	1,637 (24.89%)	3,304 (24.64%)	2,220 (23.7%)

Table S4: (Weighted) sample characteristics by number of interviews in study period. "Excluded" includes participants with fewer than 2 interviews with complete data.

			Interviews				
	Variable	Excluded	2	3	4	5	6
	n	20,451.15 (35.33%)	7,242.46 (12.51%)	4,382.86 (7.57%)	5,460.68 (9.43%)	12,215.86 (21.1%)	8,131.54 (14.05%)
Age (grouped)	18-34	7,735.96 (37.83%)	2,115.69 (29.21%)	1,176.99 (26.85%)	942.33 (17.26%)	1,549.09 (12.68%)	1,163.23 (14.31%)
	35-49	5,931.50 (29%)	2,208.02 (30.49%)	1,242.60 (28.35%)	1,484.94 (27.19%)	2,941.20 (24.08%)	1,753.41 (21.56%)
	50-64	4,278.29 (20.92%)	1,886.24 (26.04%)	1,198.47 (27.34%)	1,720.50 (31.51%)	4,132.45 (33.83%)	2,797.15 (34.4%)
	65+	2,505.40 (12.25%)	1,032.51 (14.26%)	764.80 (17.45%)	1,312.90 (24.04%)	3,593.13 (29.41%)	2,417.75 (29.73%)
Country of residence	England	17,432.69 (85.24%)	6,254.86 (86.36%)	3,474.93 (79.28%)	4,157.58 (76.14%)	10,199.19 (83.49%)	7,091.97 (87.22%)
	Scotland	1,697.04 (8.3%)	505.35 (6.98%)	359.39 (8.2%)	484.67 (8.88%)	1,077.60 (8.82%)	752.32 (9.25%)
	Wales	588.10 (2.88%)	319.79 (4.42%)	417.02 (9.51%)	705.35 (12.92%)	681.76 (5.58%)	123.00 (1.51%)
	Northern Ireland	733.31 (3.59%)	162.46 (2.24%)	131.52 (3%)	113.08 (2.07%)	257.31 (2.11%)	164.25 (2.02%)
Ethnicity	White	17,002.39 (83.14%)	6,310.17 (87.13%)	3,871.85 (88.34%)	5,003.51 (91.63%)	11,280.00 (92.34%)	7,447.99 (91.59%)
	Non-White	3,448.76 (16.86%)	932.29 (12.87%)	511.02 (11.66%)	457.17 (8.37%)	935.87 (7.66%)	683.55 (8.41%)
Gender	Male	10,581.48 (51.74%)	3,297.29 (45.53%)	2,077.74 (47.41%)	2,777.80 (50.87%)	6,347.69 (51.96%)	4,027.50 (49.53%)
	Female	9,869.66 (48.26%)	3,945.17 (54.47%)	2,305.13 (52.59%)	2,682.88 (49.13%)	5,868.17 (48.04%)	4,104.03 (50.47%)
Household income	£90k+	1,678.51 (8.21%)	648.43 (8.95%)	339.22 (7.74%)	384.49 (7.04%)	874.31 (7.16%)	704.85 (8.67%)
	£60k - £90k	2,468.68 (12.07%)	919.42 (12.69%)	574.61 (13.11%)	649.87 (11.9%)	1,340.98 (10.98%)	1,029.57 (12.66%)
	£30k - £60k	6,131.02 (29.98%)	2,322.15 (32.06%)	1,437.69 (32.8%)	1,822.21 (33.37%)	3,862.46 (31.62%)	2,658.74 (32.7%)
	£16k - £30k	5,518.62 (26.98%)	1,843.43 (25.45%)	1,095.08 (24.99%)	1,543.83 (28.27%)	3,570.31 (29.23%)	2,298.14 (28.26%)
	<£16k	4,654.31 (22.76%)	1,509.03 (20.84%)	936.25 (21.36%)	1,060.27 (19.42%)	2,567.80 (21.02%)	1,440.24 (17.71%)
Marital status	Single	5,471.43 (26.75%)	1,733.23 (23.93%)	876.25 (19.99%)	949.11 (17.38%)	1,996.54 (16.34%)	1,428.00 (17.56%)
	Divorced/Widowed	1,944.70 (9.51%)	762.72 (10.53%)	466.93 (10.65%)	753.28 (13.79%)	1,859.81 (15.22%)	1,131.50 (13.91%)
	In relationship but living apart	1,951.81 (9.54%)	620.33 (8.57%)	435.96 (9.95%)	376.98 (6.9%)	648.73 (5.31%)	495.85 (6.1%)
	Cohabiting with partner	11,083.21 (54.19%)	4,126.18 (56.97%)	2,603.73 (59.41%)	3,381.31 (61.92%)	7,710.79 (63.12%)	5,076.19 (62.43%)
Overcrowded accommodation	Not Overcrowded	17,494.06 (85.54%)	6,537.21 (90.26%)	4,044.02 (92.27%)	5,165.78 (94.6%)	11,723.88 (95.97%)	7,854.21 (96.59%)
	Overcrowded	2,957.09 (14.46%)	705.25 (9.74%)	338.85 (7.73%)	294.90 (5.4%)	491.98 (4.03%)	277.33 (3.41%)
Highest qualification	Postgraduate	2,534.98 (12.4%)	990.11 (13.67%)	650.91 (14.85%)	798.15 (14.62%)	1,670.10 (13.67%)	1,482.21 (18.23%)

			Interviews				
	Variable	Excluded	2	3	4	5	6
	Undergraduate	3,752.06 (18.35%)	1,511.49 (20.87%)	974.02 (22.22%)	1,187.31 (21.74%)	2,577.91 (21.1%)	1,858.39 (22.85%)
	A-Level or Vocational	7,121.88 (34.82%)	2,538.01 (35.04%)	1,456.83 (33.24%)	1,775.41 (32.51%)	3,978.84 (32.57%)	2,498.23 (30.72%)
	GCSE or Lower	7,042.23 (34.43%)	2,202.85 (30.42%)	1,301.11 (29.69%)	1,699.81 (31.13%)	3,989.02 (32.65%)	2,292.71 (28.2%)
Employment status	Employed	13,729.49 (67.13%)	4,977.61 (68.73%)	2,875.70 (65.61%)	3,375.24 (61.81%)	6,837.39 (55.97%)	4,578.17 (56.3%)
	Inactive	6,052.50 (29.59%)	2,115.68 (29.21%)	1,450.17 (33.09%)	2,017.37 (36.94%)	5,189.73 (42.48%)	3,453.88 (42.48%)
	Unemployed	669.16 (3.27%)	149.18 (2.06%)	56.99 (1.3%)	68.08 (1.25%)	188.74 (1.55%)	99.48 (1.22%)
Household tenure	Own Outright	3,937.05 (19.25%)	1,754.83 (24.23%)	1,179.03 (26.9%)	1,822.96 (33.38%)	4,844.29 (39.66%)	3,353.44 (41.24%)
	Own Mortgage	6,560.96 (32.08%)	2,553.18 (35.25%)	1,512.65 (34.51%)	1,846.73 (33.82%)	3,712.01 (30.39%)	2,345.08 (28.84%)
	Rent	9,953.13 (48.67%)	2,934.46 (40.52%)	1,691.19 (38.59%)	1,790.99 (32.8%)	3,659.56 (29.96%)	2,433.02 (29.92%)

Table S5: Association between total adversity worries or stressors by mental health outcome, fixed effects models including interaction with SEP (continuous measure derived from confirmatory factor analysis)

Variable	GAD-7		PHQ-9	
	Worries	Experience	Worries	Experience
Main Effect	0.069<br>[0.064, 0.074]	0.057<br>[0.043, 0.071]	0.054<br>[0.049, 0.06]	0.065<br>[0.05, 0.08]
Interaction with SEP	-0.003<br>[-0.009, 0.002]	0.001<br>[-0.013, 0.014]	0<br>[-0.005, 0.005]	0.006<br>[-0.008, 0.02]

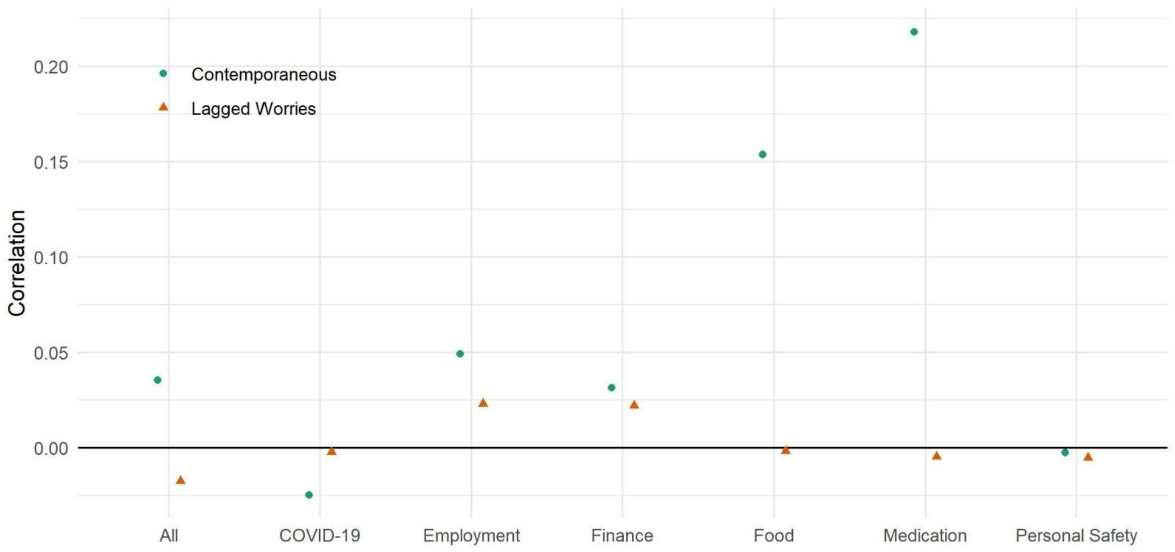

Figure S1: Correlation between adversity experience and adversity worries measures. Each measure is demeaned at the participant level given our use of fixed effects models. "Contemporaneous" refers to experiences and worries measured at the same round of data collection. "Lagged worries" refers to the correlation between adversity experiences and lagged adversity worries, and so tests whether worries are followed by adversity experiences in the short term.

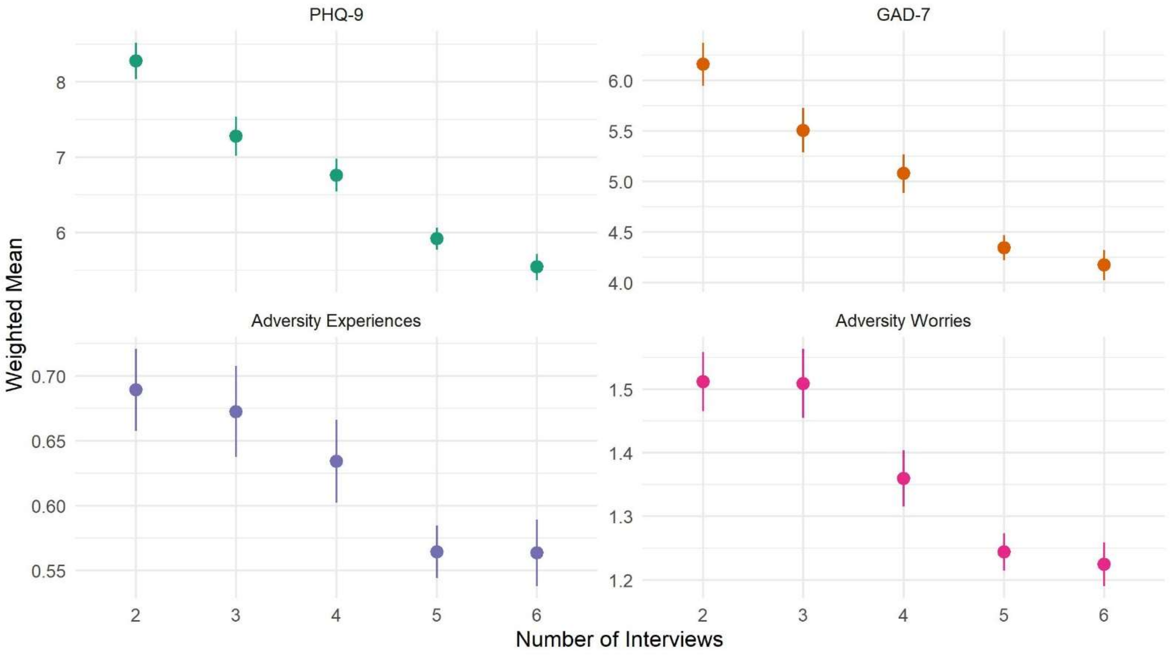

Figure S2: Weighted average GAD-7, PHQ-9 and adversity experiences and worries by number of follow-ups. Derived from linear regression models with standard errors clustered at participant level.

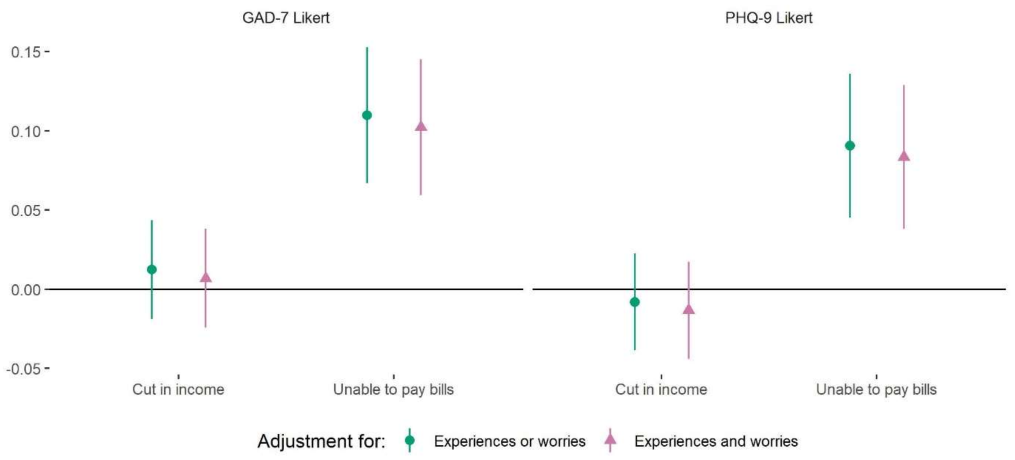

Figure S3: Associations between GAD-7 and PHQ-9 and experiencing cut in income or reporting being unable to pay bills. Results derived from fixed effects models

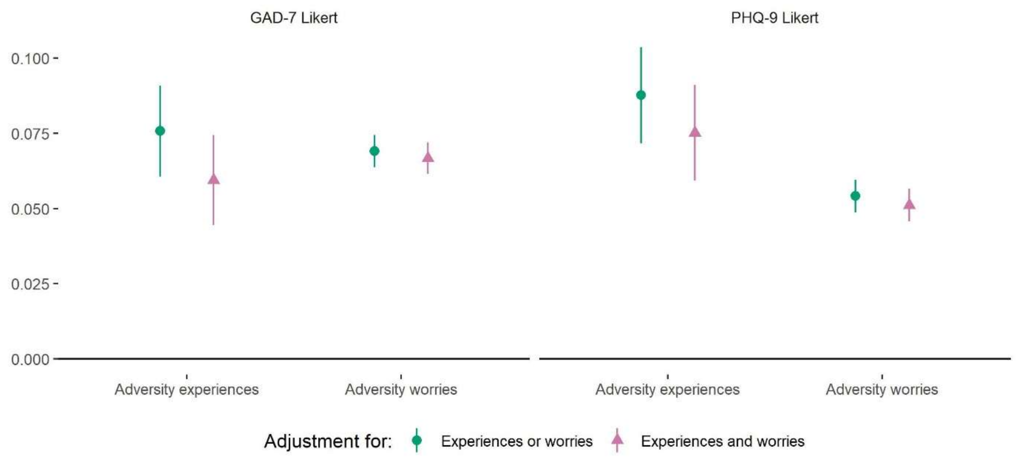

Figure S4: Associations between GAD-7 and PHQ-9 Likert Scores and increase in total stressors and total adversities, using inability to pay bills as financial adversity experience. Results derived from fixed effects models

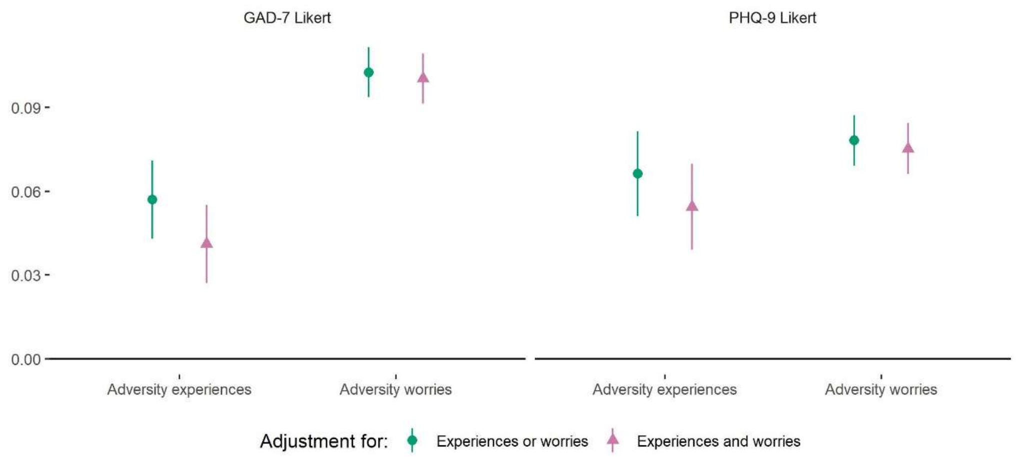

Figure S5: Associations between GAD-7 and PHQ-9 Likert Scores and increase in total stressors and total adversities, using significant stressors only to define worries. Derived from fixed effects models.

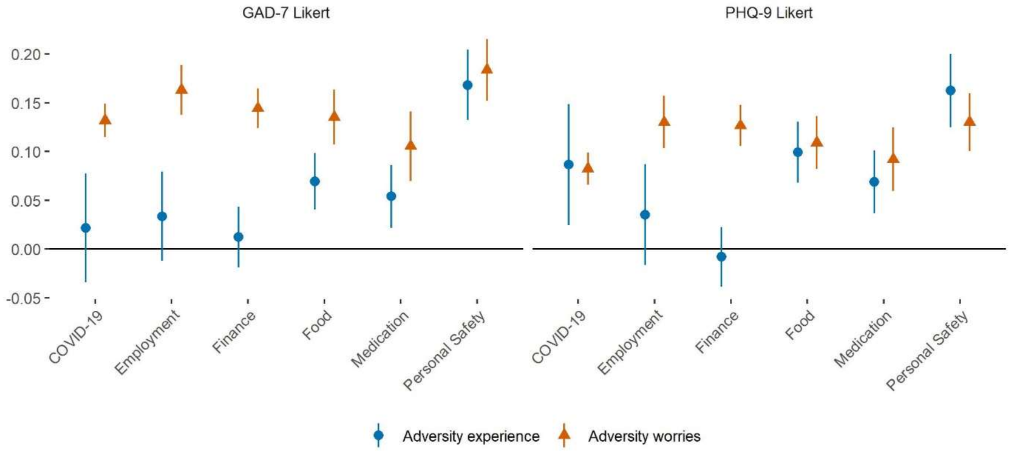

Figure S6: Association between change in GAD-7 and PHQ-9 scores and occurrence of adversity or worry about adversity, using significant stressors only to define worries. Derived from fixed effects models

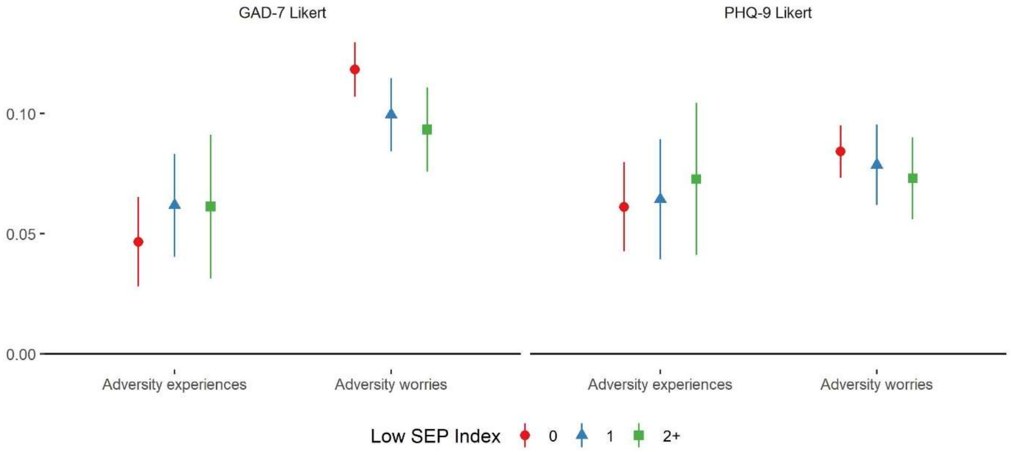

Figure S7: Associations between GAD-7 and PHQ-9 Likert Scores and increase in total adversity experiences and worries by SEP, using significant stressors only to define worries. Derived from fixed effects models

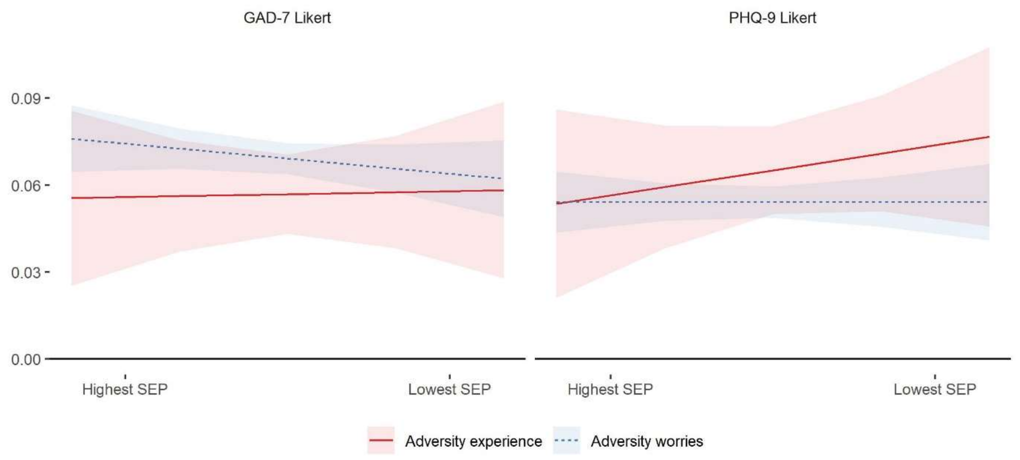

Figure S8: Associations between GAD-7 and PHQ-9 Likert Scores and increase in total adversity experiences and worries by SEP from confirmatory factor analysis, derived from fixed effects models
